# ASO Author Reflections: Intraoperative Nomograms Based on One-Step Nucleic Acid Amplification

**DOI:** 10.1245/s10434-018-6950-3

**Published:** 2018-10-26

**Authors:** Kenzo Shimazu, Shinzaburo Noguchi

**Affiliations:** Department of Breast and Endocrine Surgery, Osaka University Graduate School of Medicine, 2-2-E10 Yamadaoka, Suita-shi, Osaka 565-0871 Japan

## Past

The American College of Surgeons Oncology Group (ACOSOG) Z0011 trial has demonstrated that completion axillary lymph node dissection (cALND) can be avoided for one or two sentinel lymph nodes (SLN)-positive breast cancer patients receiving breast-conserving therapy.^1^ Avoidance of cALND is expected to result in less arm morbidity but makes unavailable the information about the total number of axillary lymph node (ALN) metastasis, which is very important for decision making for the adjuvant chemotherapeutic regimens and the indication for regional LN irradiation. Besides, the International Breast Cancer Study Group (IBCSG) 23-01 trial has demonstrated that cALND can be avoided for one or two SLN micrometastasis-positive breast cancer patients receiving breast-conserving therapy or mastectomy.^2^ It should be noticed that, in this trial, SLNs were examined meticulously, i.e., entire sectioning of each SLN at 50–200-μm intervals, which would be very difficult to perform intraoperatively in a routine practice. Therefore, for SLN-positive patients who received breast-conserving therapy and no cALND, the estimation of the total ALN metastasis, especially ≤ 3 or ≥ 4, seems to be important, and for those receiving mastectomy, development of a less laborious method for intraoperative determination of SLN micrometastasis seems to be important.

## Present

The one-step nucleic acid amplification (OSNA) assay is a rapid molecular procedure for the detection of metastasis in a whole LN, targeting cytokeratin 19 (CK19) messenger RNA (mRNA), which can be completed intraoperatively within 30–40 min.^3^ Our newly developed nomogram based on OSNA for the prediction of non-SLN metastasis in patients with positive SLN can be used intraoperatively and is as accurate as the conventional postoperative nomograms.^4^ We suggest the following strategy; the OSNA-based nomogram is utilized intraoperatively, and when a patient with SLN metastasis is estimated to have non-SLN metastasis at a probability of less than 13% (this cutoff value derives from IBCSG 23-01 trial^4^), the SLN metastasis is considered to be “micrometastasis” in terms of the probability of non-SLN metastasis, and thus cALND is avoided regardless of the type of breast surgery (breast-conserving surgery or mastectomy). In addition, estimation of probability of ≥ 4 total ALN metastasis is possible with this nomogram, which would help to decide the adjuvant chemotherapeutic regimens as well as the indication of regional LN irradiation for the SLN-positive patients receiving breast-conserving therapy without cALND.

## Future

Because the incidence of ALN metastasis can differ depending on subtypes of breast tumors, ideally nomograms specified to each subtype need to be constructed in the future. Such nomograms would more accurately predict non-SLN metastasis and would be more useful in the decision making as to the indication for cALND or postoperative therapies. Moreover, because an entire SLN is examined by OSNA unlike a routine pathology, which examines only a few representative sections from each SLN, micrometastasis is less likely to be missed by OSNA than pathology. Therefore, “OSNA-negative” is more reliable than “pathology-negative,” suggesting a possibility that “OSNA-negative” might serve as a more reliable, favorable prognostic factor than “pathology-negative.”
